# The Impact of Different Types of Social Media Use on the Mental Health of UK Adults: Longitudinal Observational Study

**DOI:** 10.2196/56950

**Published:** 2024-10-30

**Authors:** Yue Yu, Jennifer Dykxhoorn, Ruth Plackett

**Affiliations:** 1 Department of Epidemiology and Public Health University College London London United Kingdom; 2 Division of Psychiatry University College London London United Kingdom; 3 Research Department of Primary Care and Population Health University College London London United Kingdom

**Keywords:** social media, mental health, depression, anxiety, mental disorders, cohort studies, United Kingdom, longitudinal observational study

## Abstract

**Background:**

Previous studies have explored the association between social media use and mental health among adolescents. However, few studies using nationally representative longitudinal data have explored this relationship for adults and how the effect might change depending on how people use social media.

**Objective:**

This study investigated the longitudinal relationship between the frequency of viewing and posting on social media and mental health problems among UK adults.

**Methods:**

This study included 15,836 adults (aged 16 years and older) who participated in Understanding Society, a UK longitudinal survey. Social media use was measured with questions about the frequency of viewing social media and posting on social media in Understanding Society Wave 11 (2019-2021). We explored viewing and posting separately, as well as a combined exposure: (1) high viewing, high posting; (2) high viewing, low posting; (3) low viewing, high posting; and (4) low viewing, low posting. Mental health problems were measured in Wave 12 (2020-2022) using the General Health Questionnaire (GHQ-12), a validated scale for identifying symptoms of common mental health problems, where higher scores indicated more mental health problems (0 to 36). Unadjusted and adjusted linear regression models were estimated for viewing social media and posting on social media, adjusting for the baseline GHQ score, gender, age, ethnicity, employment, and education. We found no evidence for effect modification by gender and age so overall associations were reported.

**Results:**

In our adjusted models, we found no evidence of an association between the frequency of viewing social media and mental health problems in the following year. We found that adults who posted daily on social media had more mental health problems than those who never posted on social media, corresponding to a 0.35-point increase in GHQ score (β=0.35, 95% CI 0.01-0.68; *P*=.04). When we considered both social media behaviors, we found that those who frequently viewed and posted on social media scored 0.31 points higher on the GHQ score (β=0.31, 95% CI 0.04-0.58; *P*=.03) in the following year compared to those who rarely viewed or posted on social media.

**Conclusions:**

We found that a high frequency of posting on social media was associated with increased mental health problems a year later. However, we did not find evidence of a similar association based on the frequency of viewing social media content. This provides evidence that some types of active social media use (ie, posting) have a stronger link to mental health outcomes than some types of passive social media use (viewing). These results highlighted that the relationship between social media use and mental health is complex, and more research is needed to understand the mechanisms underlying these patterns to inform targeted interventions and policies.

## Introduction

In the United Kingdom, around 1 in 4 people experience mental health problems each year [1], and improving mental health is a public health priority [2]. The growth of social media in recent decades has caused speculation that the increasing prevalence of mental health problems could be linked to social media use [3,4]. While social media use has become an integral part of everyday life for many people, social media users can interact with these internet-based tools in different ways. Individuals generally engage with social media passively or actively. Passive social media use includes browsing content and viewing posts, while active social media use includes creating public posts or sending private messages to friends, family, and others, and many engage in both passive and active social media use [5].

Due to the hypothesized link between social media use and mental health problems, several studies have explored this relationship, but the findings to date are mixed. Some studies have found a small association between social media use and increased mental health problems, such as anxiety, depressive symptoms, and psychological distress, particularly for female participants [6-8]. Other studies have shown that social media can benefit mental health, as it allows people to interact with others, can strengthen social connectedness [9], and reduce loneliness and social isolation [6,10]. Other studies have found no evidence of an association between social media use and mental health problems [11-13].

Most of the existing literature has focused on the overall time spent on social media, not specifically considering the types of social media use [6,14,15]. Some studies that have distinguished between active and passive social media use have suggested that active use can elicit social support and positive feedback, having a positive effect on mental health, whereas passive use might be more harmful to mental health than active use because it encourages upward social comparison and envy [14]. For example, some studies have concluded that active social media use, defined as posting or interacting with other’s content, was related to less loneliness and less mental health problems, while passive social media was associated with increased loneliness and more mental health problems, especially for females [16-18]. However, a systematic review found that overall existing studies do not support the hypothesis that active social media use is more beneficial for mental health than passive use as these behaviors were often crudely measured with varying definitions and measurements of active and passive use [5].

Many of the aforementioned studies were conducted on adolescents and young adults [6,15,17], and the evidence for adult population remains limited [10,19,20]. Importantly, different age groups have been shown to use different social media platforms and may have different patterns of social media use. For example, data have indicated that a majority of younger adults use Instagram and Snapchat the most but older adults usually use Facebook and YouTube [21]. Evidence has also shown that younger age groups use social media more for self-presentation, but older adults use social media more for enjoyment or keeping up with family and friends [22], which may have different implications for mental health. For example, younger adults may experience more adverse mental health effects from social media use due to social comparison [23], while older adults may be less likely to engage in social comparison and instead benefit from enhanced social connectedness and reduced loneliness resulting from keeping in touch with family and friends [24].

Most previous studies in this area have explored the relationship between social media use and mental health using cross-sectional designs with nonrepresentative data [6,25]. This restricts what temporal conclusions can be drawn about the relationship and how generalizable the findings are to the general population. Some longitudinal studies have been conducted in the United Kingdom, but many suggested nonsignificant associations for adolescents and only focused on time spent on social media rather than the different types of activities on social media [11,26,27]. This study aimed to use nationally representative data to examine the longitudinal association between viewing social media content and posting on social media and mental health problems in UK adults. We expected to find an association between high levels of viewing and posting and mental health problems in the following year but expected there to be stronger evidence for viewing social media (passive use) and increased mental health problems than posting (active use). We also expected the effects to be different according to different age groups and gender. For example, the effect of social media use on mental health will be smaller among older adults than young adults but stronger in females than in males, based on previous literature. This study will provide evidence of the temporal relationship between social media use and mental health problems, as well as help to inform strategies to mitigate the risks of different types of social media use in adults.

## Methods

### Study Population

This study used data from Understanding Society (USoc), a longitudinal household survey, and a representative sample of the UK population [28]. All adults (aged 16 years or older) who completed the survey in Wave 11 (2019-2021; baseline) and Wave 12 (2020-2022; follow-up) were included in our sample. The flowchart in Multimedia Appendix 1 displays the sample selection process.

### Exposures

Two social media exposures were measured at baseline: (1) frequency of viewing social media, and (2) frequency of posting on social media. Frequency of viewing social media was measured by the question, “How often do you use the internet for personal use by looking at content on social media or websites and apps (eg, looking at text, images, and videos on Facebook, Twitter, Instagram)?” Frequency of posting was measured by the question, “How often do you use the internet for personal use by posting content on social media or websites and apps (eg, posting text, images, videos on Facebook, Twitter, Instagram)?”; including posting to private accounts. The frequency for both questions was categorized as: (1) every day, (2) several times a week, (3) several times a month, (4) once a month, (5) less than once a month, and (6) never. Participants who had indicated that they did not have access to or use the internet were recorded as “never” for both frequency of viewing and frequency of posting. Those who reported “refusal” and “don’t know” were counted as “missing.”

We created a combined variable to capture both types of social media use. Participants were categorized as having a high frequency of viewing and posting if they reported “Every day,” and “Several times a week,” and those who reported “Several times a month,” “Once a month,” “Less than once a month,” and “Never” were classified as low frequency. Participants were assigned to one of the following groups: (1) high viewing, high posting; (2) high viewing, low posting; (3) low viewing, high posting; and (4) low viewing, low posting.

### Outcome

Mental health problems were measured in Wave 12 using the General Health Questionnaire (GHQ-12). The GHQ-12 includes 12 questions that assess psychological distress and symptoms of anxiety or depression over the past weeks. We used the recommended Likert scoring method [29], where each question is scored from 0 to 3, resulting in a total score from 0 to 36. Higher GHQ-12 scores indicate higher levels of psychological distress and more mental health problems.

### Confounders

We included gender (female and male), age group (16-29, 30-44, 45-59, and 60 and older), education (6 levels: higher degree, 1st degree or equivalent, other higher degree, A level, General Certificate of Secondary Education/O level, and other qualification), employment (employed and not in paid employment), and ethnicity (White and all other ethnic groups combined) as confounders in this analysis. All confounders were measured at baseline, and we also adjusted for mental health problems at baseline (baseline GHQ-12 score).

### Effect Modification

We tested for effect modification by gender and age group. As we did not find evidence for effect modification, we reported overall estimates.

### Missing Data

Any exposure, covariate, or outcome that was missing data, as well as any responses of “don’t know,” or “refusal,” were imputed. All variables in the regression model were used in the imputation model. Age, gender, and ethnicity had no missing data, so were included in the imputation models as auxiliary variables. We created 20 imputed datasets using multiple imputations with chained equations. Our primary analysis was based on the imputed data, which was compared to a sensitivity analysis of complete cases (Multimedia Appendix 2).

### Weighting

To account for the household clustered, stratified survey design, correct for nonresponse and loss-to-follow-up, we used the longitudinal weight from Wave 12 provided by USoc. This allowed our analyses to better represent the adult population in the United Kingdom and to improve the reliability and validity of our findings. We also excluded from the analysis any participants who had a zero weight due to sample design or fieldwork issues in USoc, as the weighted analysis compensated for these cases being missing. We used the svy command in Stata (version 17; StataCorp LLC) to account for the complex survey design and the clustering at individual and area levels.

### Statistical Analysis

We conducted unadjusted and adjusted linear regression on the imputed analytic sample. Model 1 (unadjusted) investigated the associations between social media use and mental health, model 2 included the adjustment for baseline mental health problems, and model 3 (fully adjusted) additionally adjusted for gender, age, education, employment, and ethnicity. These models were estimated for all three exposures: (1) frequency of viewing social media, (2) frequency of posting on social media, and (3) social media viewing and posting frequency. All analysis was conducted using Stata.

### Ethical Considerations

This study involved secondary data analysis of the USoc dataset, which had already received ethical approval, and this approval stated that secondary analyses did not require additional approval. The University of Essex Ethics Committee has approved all data collection on USoc main study and innovation panel waves (ethics approval ETH1920-0123 for Wave 12). Participants of USoc provided informed consent for the use of their data in future primary or secondary research contexts. All data used in this study were anonymized by USoc prior to being accessed, ensuring that no identifying information was available to the researchers. Participants were informed at the time of data collection that their data would be used anonymously and for research purposes only, with no analysis being conducted at the individual level. Participants were compensated for taking part in USoc.

## Results

### Descriptive Analysis

There were 15,836 participants included in our analytic sample, with an average age of 53 (SD 18.08) years (Table 1). There were more female (n=9105, 57.5%) participants than male participants, and most of the sample was of White ethnicity (n=13,978, 88.3%). Just over half (n=8298, 52.4%) of the sample were employed, while the rest were not in paid employment. More than half of the participants (n=8761, 55.3%) viewed social media every day at baseline and only 15.4% (n=2433) of participants posted on social media every day. Around a fifth of participants (n=3504, 22.1%) have never viewed social media, and a larger percentage of participants (n=5767, 36.4%) never posted on social media.

**Table 1:** Descriptive statistics of the sample in terms of the key variables (N=15,836).

Variables		Value, n (%)	Mean (SD)
**Age (in years)**			53.50 (18.1)
	16-29	2062 (13)	
	30-44	2621 (16.6)	
	45-59	4556 (28.8)	
	60 and older	6597 (41.7)	
	Missing	0 (0)	
**Sex**			N/A^a^
	Male	6731 (42.5)	
	Female	9105 (57.5)	
	Missing	0 (0)	
**Education**			N/A
	Higher degree	1739 (11)	
	1st degree or equivalent	2491 (15.7)	
	Other higher degree	1546 (9.8)	
	A level^b^	1236 (7.8)	
	GCSE^c^/O level	2798 (17.7)	
	Other qualification	1200 (7.6)	
	Missing	4826 (30.5)	
**Employment**			N/A
	Employed	8298 (52.4)	
	Not in paid employment	7509 (47.4)	
	Missing	29 (0.2)	
**Ethnicity**			N/A
	White background	13,978 (88.3)	
	All other ethnic groups combined	1858 (11.7)	
	Missing	0 (0)	
**Baseline GHQ-12 score (wave 11)**			
	Score	N/A	11.40 (5.45)
	Missing	142 (0.9)	N/A
**Follow-up GHQ-12 score (wave 12)**			
	Score	N/A	11.74 (5.67)
	Missing	152 (1.0)	N/A
**Frequency of viewing social media**			N/A
	Everyday	8761 (55.3)	
	Several times a week	1990 (12.6)	
	Several times a month	700 (4.4)	
	Once a month	269 (1.7)	
	Less than once a month	579 (3.7)	
	Never	3504 (22.1)	
	Missing	33 (0.2)	
**Frequency of posting on social media**			N/A
	Everyday	2433 (15.4)	
	Several times a week	2217 (14)	
	Several times a month	2112 (13.3)	
	Once a month	998 (6.3)	
	Less than once a month	2277 (14.4)	
	Never	5767 (36.4)	
	Missing	32 (0.2)	
**Frequency of viewing and posting on social media**			N/A
	Low viewing, low posting	4970 (31.4)	
	Low viewing, high posting	75 (0.5)	
	High viewing, low posting	6175 (39)	
	High viewing, high posting	4569 (28.9)	
	Missing	47 (0.3)	

^a^N/A: not applicable.

^b^A level: advanced level qualifications.

^c^GCSE: General Certificate of Secondary Education.

A large proportion of participants (n=6175, 39%) viewed social media frequently but posted less frequently, 31.4% (n=4970) of participants viewed and posted on social media both at low frequency, 28.9% (n=4569) of the participants viewed and posted both at high frequency, it is unusual (n=75, 0.5%) for participants to view social media less frequently but post frequently. The mean GHQ score at baseline was 11.40 (SD 5.45) and was 11.74 (SD 5.67) at follow-up.

### Imputation

The proportion of missing data at baseline can be found in Table 1. There was no missing data for gender, age, and ethnicity, but a high proportion of missing data for education (n=4826, 30.5%), and less than 1% missing for employment, GHQ scores, and social media variables (Table 1).

### Effect Modification

We tested for the presence of effect modification by gender and age but did not find evidence for effect modification for either variable once we adjusted for baseline mental health problems and other confounders (Multimedia Appendices 3 and 4). Therefore, both gender and age were included as confounders and were adjusted with other covariates in the fully adjusted model.

### Linear Regression Analysis

#### Overview

Table 2 shows the results of the linear regression models estimating the association between social media exposures and later mental health problems.

**Table 2:** Linear regression model results for the association between types of social media use and mental health problems (N=15,836).

Variables		Value, n (%)	Model 1: unadjusted (crude association)			Model 2: adjusted for baseline GHQ^a^			Model 3: fully adjusted^b^		
			β	95% CI	*P* value	β	95% CI	*P* value	β	95% CI	*P* value
**Frequency of viewing social media**											
	Never	3517 (22.2)	reference	reference	reference	reference	reference	reference	reference	reference	reference
	Less than once a month	579 (3.66)	.25	–0.28 to 0.78	.35	.00089	–0.46 to 0.46	.99	–.0003	–0.46 to 0.46	.99
	Once a month	271 (1.71)	.94	0.04 to 1.83	.04	–.12	–0.81 to 0.57	.73	–.16	–0.85 to 0.53	.64
	Several times a month	701 (4.43)	.86	0.30 to 1.41	.003	.49	0.04 to 0.93	.03	.43	–0.07 to 0.87	.05
	Several times a week	1997 (12.6)	.77	0.35 to 1.19	<.001	.36	–0.01 to 0.73	.06	.23	–0.13 to 0.58	.22
	Every day	8771 (55.4)	1.34	1.05 to 1.64	<.001	.53	0.29 to 0.76	<.001	.12	–0.13 to 0.38	.34
**Frequency of posting on social media**											
	Never	5781 (36.5)	reference	reference	reference	reference	reference	reference	reference	reference	reference
	Less than once a month	2279 (14.4)	.69	0.33 to 1.05	<.001	.26	–0.04 to 0.56	.09	.06	–0.24 to 0.36	.69
	Once a month	1001 (6.32)	.57	0.10 to 1.04	.02	.20	–0.22 to 0.61	.35	–.09	–0.50 to 0.32	.66
	Several times a month	2118 (13.4)	.90	0.52 to 1.28	<.001	.39	0.08 to 0.71	.01	.10	–0.22 to 0.42	.54
	Several times a week	2222 (14)	1.39	1.02 to 1.77	<.001	.71	0.41 to 1.01	<.001	.38	0.07 to 0.69	.02
	Every day	2435 (15.4)	1.56	1.16 to 1.95	<.001	.73	0.41 to 1.06	<.001	.35	0.01 to 0.68	.04
**Frequency of viewing and posting on social media**											
	Low viewing, low posting	4991 (31.5)	reference	reference	reference	reference	reference	reference	reference	reference	reference
	Low viewing, high posting	77 (0.50)	.93	–0.58 to 2.44	.23	.95	–.16 to 2.06	.09	.76	–0.38 to 1.90	.19
	High viewing, low posting	6188 (39.1)	.74	0.46 to 1.01	<.001	.26	0.03 to 0.49	.03	–.03	–0.26 to 0.20	.81
	High viewing, high posting	4580 (28.9)	1.53	1.21 to 1.85	<.001	.71	0.46 to 0.97	<.001	.31	0.04 to 0.58	.03

^a^GHQ: General Health Questionnaire.

^b^Covariates: gender, age, employment, education, and ethnicity.

#### Viewing Social Media

In the unadjusted model, adults who viewed social media once a month or more had higher GHQ scores a year later than those who never viewed social media content. The largest increase was observed in those who viewed social media every day, where the mean GHQ scores were 1.34 points higher (95% CI 1.05-1.64; *P*<.001) than those who never viewed social media content. Once we adjusted for baseline mental health problems, those who viewed social media every day, or several times a month continued to have elevated GHQ scores compared to those who never viewed social media. However, once we adjusted for all confounders, this association was fully attenuated, and we found no evidence of increased mental health problems based on the frequency of viewing social media content.

#### Posting on Social Media

In the unadjusted model, participants who ever posted on social media had higher GHQ scores a year later than those who never posted, with the largest increase in those who posted every day (β=1.56, 95% CI 1.16-1.95; *P*<.001), with a similar increase in those who posted several times a week (β=1.39, 95% CI 1.02-1.77; *P*<.001). Following adjustment for the baseline GHQ, those who posted every day, several times a week, or several times a month continued to have higher GHQ scores than those who never posted. Finally, in the fully adjusted model, those who posted the most frequently (every day or several times a week) had elevated GHQ scores, but the magnitude of this increase had attenuated (every day: β=0.35, 95% CI 0.01-0.68; *P*=.04; several times a week: β=0.38, 95% CI 0.066-0.69; *P*=.02).

#### Viewing and Posting on Social Media

When we considered both social media exposures simultaneously, we found that those with a high frequency of both viewing and posting had an increased GHQ score across all models, although the magnitude of the association attenuated with each adjustment (unadjusted: β=1.53; 95% CI 1.21-1.85; *P*<.001; baseline GHQ adjusted: β=0.71, 95% CI 0.46-0.97; *P*<.001; fully adjusted: β=0.31, 95% CI: 0.04-0.58; *P*=.03). We found higher GHQ scores in the unadjusted and partially adjusted models for those who frequently viewed social media but posted infrequently, however, this association fully attenuated in the adjusted model (Table 2).

### Complete Case Sensitivity Analysis

We repeated the linear regression models in the complete case sample (n=10,869), finding similar patterns (Multimedia Appendix 2). The complete case analysis also found no evidence of an association between the frequency of viewing social media and GHQ in the fully adjusted model, some evidence of an association between higher frequency of posting and higher GHQ scores, but the magnitude of effect attenuated with each adjustment. There was no evidence of an association between both viewing and posting frequency on social media and GHQ scores later.

## Discussion

### Principal Findings

This longitudinal study investigated the relationship between different types of social media use and later mental health problems in UK adults. We found no evidence for an association between the frequency of viewing social media content and later mental health problems. In contrast, we found that individuals who posted social media content several times a week or more had increased mental health problems at follow-up compared to those who never posted, even after accounting for baseline mental health and a range of covariates. This suggested that some types of active use of social media, specifically posting, might have a more negative effect on later mental health than passive use of social media. When looking at both behaviors, we found that individuals who frequently viewed and posted on social media had increased mental health problems when compared to individuals who rarely viewed or posted on social media.

### Comparisons With Previous Studies

The results of this study contradicted some previous studies exploring the links between types of social media use and mental health. For example, a longitudinal study found an association between active social media use and reduced depressive symptoms for female adolescents [18]. Their study measured active use by measuring how often an adult posted messages or pictures or videos about themselves or others in the past 6 months. A cross-sectional study based on US adults aged 18-49 years old found evidence that a higher frequency of passive social media use related to increased depressive symptoms, while active use was associated with reduced depressive symptoms [20]. Their study defined active use as how often they liked or shared or posted and commented on others’ content. In contrast, we did not find evidence of an association between a higher level of passive social media use and more mental health problems, but we concluded that a higher frequency of active social media use was related to more mental health problems a year later, and this did not vary by gender. The differences in how social media activities were measured could account for the inconsistency between these conclusions. Our study assessed active use of social media by asking how often somebody posted messages or pictures or videos on social media, which may not have been as comprehensive as some of the activities included in the other studies and may account for the different findings. To enable better comparison of findings, future research should use more specific and consistent measures of active and passive use. The discrepancies between these studies and our findings could also be due to the different age groups included in the analysis and our analysis was the only analysis to be conducted with adults on longitudinal nationally representative data.

Our findings were more consistent with a cross-sectional study that concluded that for adults, active social media use was associated with severe anxiety and stress but not severe depression, whereas, passive social media use showed no significant association with mental health problems [30]. Like our study, this study measured active use by how often a person posted or commented on posts, but the analysis was not longitudinal or conducted on nationally representative data. Our conclusion that more frequent posting on social media was related to poorer mental health a year later, aligned with a recent longitudinal study conducted by Lowthian et al [16] that explored social media use of adolescents in the COVID-19 pandemic. They distinguished between 2 types of active social media use: posting and messaging with internet-only friends, their findings suggested that both active social media use and passive social media use were associated with poorer mental well-being. However, “avid” users who were defined as having the highest communication frequency with people they did not know on social media had the poorest mental health outcomes, compared to those who used social media to speak with friends and family and for social or political engagement. This suggests that some public active social media activities, like messaging strangers on social media or posting publicly, may be more harmful to some people than other active uses like private messaging with friends and family or engaging in social or political activities. We also found that adults who both frequently viewed and posted on social media have more mental health problems a year later than those who rarely used social media for either purpose. This conclusion was also consistent with a longitudinal study that suggested that adolescents who frequently shared content, messaged, and browsed social media were more likely to have poorer mental health a year later than those who minimally used social media [31]. Overall, these findings suggest that both time spent on social media and type of activity are important to consider in the relationship between social media use and mental well-being and should be measured in future studies. However, we also need to measure more specific active uses of social media both public and private to get a more nuanced understanding of what kinds of activities might be harmful or helpful for our mental health and for which groups of people.

Our findings were contradictory to our hypotheses that passive use would be more harmful to adult mental health than active use, but some research has suggested why some types of active social media use may lead to poorer mental health. Posting actively on social media may have negative effects on mental health because people may post unrealistic content about themselves or because they are replacing in-person relationships with web-based relationships [30]. Some types of active use, such as messaging friends, may have positive effects on mental health as it can improve social connectedness and reduce loneliness. Further, some types of passive social media use may not have a negative impact on mental health because people may be positively comparing themselves to others or receive admiration or inspiration from others [32,33]. Theories about social media use and the relationship with mental health are still developing and further qualitative research will help to develop our understanding of the underlying mechanisms in this relationship. In addition, the effects of social media on mental health are likely to be affected by the type of content posted or viewed on social media. For example, studies have found that viewing self-harm–related images or words on social media has a range of harmful effects on people’s mental health, such as a higher level of emotional distress and more self-harming thoughts and behaviors [34]. Further research is needed to explore not only the range of activities people do on social media but also the types of content people view or post on social media and how they might affect mental health.

There was no evidence from this study showing gender or age differences in the association between social media use and mental health problems. This is despite previous literature suggesting that younger age groups and female groups might have worse mental health outcomes as a result of problematic social media use than male and older age groups [6,23,24]. Understanding the specific activities that can be helpful and harmful for mental health for different groups could help to target social media use management interventions at those who are most susceptible to the negative effects of social media use and harness some of the benefits of social media to improve mental health.

### Strengths and Weakness

USoc is a large longitudinal study providing a nationally representative sample of UK adults, making the findings from this study generalizable to the UK population. A key strength of this study was that we were able to consider a more detailed picture of social media use by examining viewing and posting frequency on social media. Given that most existing literature has only used time spent on social media as the exposure, this study provided a more comprehensive understanding of social media behaviors. This longitudinal analysis can shed light on the potential causal relationship between social media use and mental health by accounting for the temporal order of exposures and outcomes. We adjusted for mental health at baseline, to account for the reverse causality of social media on mental health that has been suggested by previous research: poor mental health conditions may drive heavier social media use [35].

It is important to consider various limitations of this study. This study was only based on two waves of data because the two main exposures were only available in these two waves. This may not be sufficient to generate conclusions about long-term trends in mental health conditions. Future studies, with longer follow-ups, would explore the long-term impacts of social media use on mental health.

The complexity of the study design raised challenges in developing accurate weights for this population. While USoc was designed (in Wave 1) to be representative of the UK population, wave nonresponse and loss-to-follow-up affect the sample in subsequent waves. In line with the recommendations from USoc, we used the provided longitudinal weights. These weights were created for monotone longitudinal response and include design weights to account for differences in selection probabilities and wave nonresponse adjustments. Where a participant missed one or more previous waves, they will receive a zero-weight in this analysis, so were dropped from this analysis. While this reduces the sample size, there was sufficient statistical power to conduct this analysis and it ensured that the results were generalizable to the UK population.

Additionally, the outcome GHQ-12 was based on 12 self-report survey questions to measure mental health problems, which may lead to memory bias as participants may have difficulty accurately recalling their mental health symptoms over a specific period before the survey, leading to inaccuracies in the scores. Hence, future studies may consider supplementing self-report measures with clinical evaluations by mental health professionals for a comprehensive understanding of participants’ mental health problems. Furthermore, this study used social media use data from Wave 11 as the exposure variables, which was collected from 2019 to 2021, when the COVID-19 pandemic was prevalent. Studies suggest that the COVID-19 outbreak largely increased social media use in many countries [36,37], which could have affected the posting frequency on social media [38]. Evidence also showed that during the pandemic, many people experienced poorer mental health due to isolation policies, illness, job loss, and other factors [39,40]. Therefore, the conclusions of this study might be less applicable to the context before or after the pandemic. We ran a sensitivity analysis, to attempt to account for the influence of COVID-19, by controlling for the year in which people took part in the study at baseline and found this did not change the results significantly.

This study offered insights into the impact of social media use on mental health in adults. Future research could further explore the impact of increased mental health problems resulting from frequent active social media use by incorporating additional measures of social functioning such as social connectedness, family relations, and leisure activities [41]. By further assessing the social functioning of participants, we could enhance the understanding of the real-life impact of the increased mental health problems observed in this study. This could then inform more targeted interventions and support strategies to improve social outcomes for adults who experienced more mental health challenges due to frequent or problematic social media use.

### Conclusions

We found that the ways in which adults use social media have a differential impact on mental health. While we found no evidence that the frequency of viewing social media content was linked to mental health outcomes, we found that frequently posting on social media was associated with increased mental health problems a year later compared to those who never posted on social media. This increase in mental health problems was also observed in those who both frequently viewed social media content and frequently posted on social media, compared to those who rarely used social media for either purpose. To help mitigate the negative impacts on adult mental health, it would be helpful to encourage people to be mindful of how they use social media, and the content they interact with, to develop healthy digital behaviors. Some types of active use, like public posting and interacting with people we do not know on social media, might have more harmful effects on mental health than actively using social media for more private uses such as messaging friends. Social media companies also have a responsibility to protect users from harmful content, which can also affect mental health, and better regulations from governments could help to ensure greater progress on social media safety.

Further research is needed to explore the range of activities people do on social media, how they might affect mental health, and which groups are most negatively affected. This could help us to understand how to harness the strengths of social media to improve mental well-being and to address the negative effects of social media use on mental health.

## Appendix

Multimedia Appendix 1 Flowchart showing the sample selection process.

Multimedia Appendix 2 Linear regression model results for the association between types of social media use and mental health problems for complete case analysis (N=10,869).

Multimedia Appendix 3 Results for effect modification for gender and age (adjusted data, imputed sample, N=15,836).

Multimedia Appendix 4 The association between posting frequency and mental health stratified by age (adjusted data, imputed sample, N=15,836).

## Abbreviations

GHQ: General Health Questionnaire
USoc: Understanding Society
