# Supplementary figures and images for: The Impact of Different Types of Social Media Use on the Mental Health of UK Adults: Longitudinal Observational Study
Source: J Med Internet Res. 2024 Oct 30;26:e56950. doi: 10.2196/56950 (PMC11561428; doi:10.2196/56950)

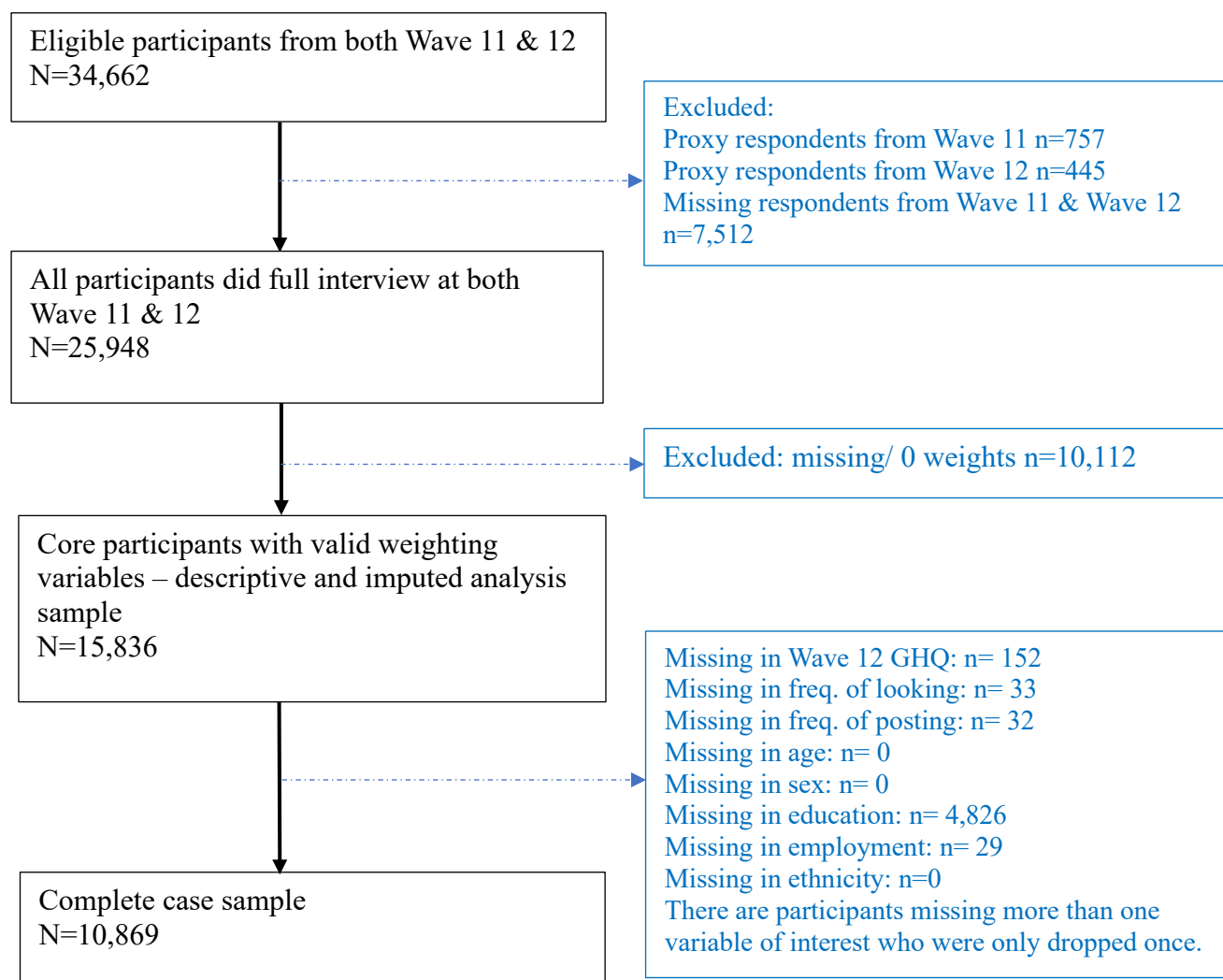

Supplement: Multimedia Appendix 1 [file jmir_v26i1e56950_app1.pdf]
