# Supplementary material for: The Impact of Different Types of Social Media Use on the Mental Health of UK Adults: Longitudinal Observational Study
Source: J Med Internet Res. 2024 Oct 30;26:e56950. doi: 10.2196/56950 (PMC11561428; doi:10.2196/56950)
Supplement: Multimedia Appendix 2 [file jmir_v26i1e56950_app2.docx]

| Variables | n (%) | Model 1: Unadjusted (crude association) | | | Model 2: Adjusted for baseline GHQ | | | Model 3: Adjusted for all covariates^1^ + baseline GHQ | | |
| --- | --- | --- | --- | --- | --- | --- | --- | --- | --- | --- |
|  |  | **β** | **95% CI** | **P value** | **β** | **95% CI** | **P value** | **β** | **95% CI** | **P value** |
| Frequency of viewing social media | | | | | | | | | | |
| *Never* | 1819 (16.7) | ref. | | | ref. | | | ref. | | |
| *Less than once a month* | 406 (3.74) | 0.34 | -0.40, 1.08 | 0.37 | 0.10 | -0.56, 0.75 | 0.77 | 0.12 | -0.54, 0.77 | 0.73 |
| *Once a month* | 180 (1.67) | 0.50 | -0.66, 1.65 | 0.40 | -0.40 | -1.35, 0.55 | 0.41 | -0.43 | -1.38, 0.52 | 0.37 |
| *Several times a month* | 472 (4.34) | 0.72 | -0.08, 1.53 | 0.08 | 0.41 | -0.25, 1.06 | 0.22 | 0.39 | -0.26, 1.04 | 0.24 |
| *Several times a week* | 1356 (12.5) | 0.68 | 0.11, 1.25 | 0.02 | 0.35 | -0.14, 0.84 | 0.17 | 0.25 | -0.24, 0.74 | 0.32 |
| *Everyday* | 6636 (61.1) | 1.19 | 0.76, 1.62 | <0.001 | 0.37 | 0.04, 0.70 | 0.03 | 0.004 | -0.36, 0.37 | 0.98 |
| Frequency of posting on social media | | | | | | | | | | |
| *Never* | 3366 (31.0) | ref. | | | ref. | | | ref. | | |
| *Less than once a month* | 1701 (15.7) | 0.45 | -0.03, 0.91 | 0.07 | 0.08 | -0.32, 0.47 | 0.70 | -0.050 | -0.45, 0.35 | 0.81 |
| *Once a month* | 734 (6.75) | 0.58 | -0.01, 1.18 | 0.06 | 0.25 | -0.24, 0.73 | 0.32 | 0.025 | -0.48, 0.53 | 0.92 |
| *Several times a month* | 1638 (15.1) | 0.72 | 0.24, 1.19 | 0.003 | 0.31 | -0.09, 0.71 | 0.13 | 0.074 | -0.34, 0.48 | 0.72 |
| *Several times a week* | 1601 (14.7) | 1.20 | 0.72, 1.68 | <0.001 | 0.69 | 0.30, 1.07 | <0.001 | 0.41 | 0.003, 0.81 | 0.05 |
| *Everyday* | 1829 (16.8) | 1.40 | 0.88, 1.91 | <0.001 | 0.61 | 0.20, 1.03 | 0.004 | 0.29 | -0.45, 0.35 | 0.20 |
| Frequency of viewing and posting on social media | | | | | | | | | | |
| Low viewing, low posting | 2828 (26.0) | ref. | | | ref. | | | ref. | | |
| Low viewing, high posting | 49 (0.5) | 0.81 | -1.58, 3.19 | 0.51 | 0.80 | -0.98, 2.57 | 0.38 | 0.64 | -1.16, 2.44 | 0.49 |
| High viewing, low posting | 4611 (42.4) | 0.59 | 0.20, 0.98 | 0.003 | 0.11 | -0.20, 0.42 | 0.49 | -0.14 | -0.47, 0.19 | 0.41 |
| High viewing, high posting | 3381 (31.1) | 1.36 | 0.93, 1.79 | <0.001 | 0.60 | 0.26, 0.94 | 0.001 | 0.23 | -0.15, 0.60 | 0.24 |
