# Supplementary material for: The Impact of Different Types of Social Media Use on the Mental Health of UK Adults: Longitudinal Observational Study
Source: J Med Internet Res. 2024 Oct 30;26:e56950. doi: 10.2196/56950 (PMC11561428; doi:10.2196/56950)
Supplement: Multimedia Appendix 3 [file jmir_v26i1e56950_app3.docx]

| Variables | P for interaction | |
| --- | --- | --- |
|  | Gender | Age |
| Frequency of viewing social media | 0.11 | 0.60 |
| Frequency of posting on social media | 0.091 | 0.050 |
