# Supplementary material for: The Impact of Different Types of Social Media Use on the Mental Health of UK Adults: Longitudinal Observational Study
Source: J Med Internet Res. 2024 Oct 30;26:e56950. doi: 10.2196/56950 (PMC11561428; doi:10.2196/56950)
Supplement: Multimedia Appendix 4 [file jmir_v26i1e56950_app4.docx]

| Variables | Age | | | | | | | | | | | |
| --- | --- | --- | --- | --- | --- | --- | --- | --- | --- | --- | --- | --- |
|  | **16-29 years old** | | | **30-44 years old** | | | **45-59 years old** | | | **60-100 years old** | | |
|  | **β** | **95% CI** | **p** | **β** | **95% CI** | **p** | **β** | **95% CI** | **p** | **β** | **95% CI** | **p** |
| Frequency of posting on social media |  | | | | | | | | | | | |
| *Never* | (reference) | | | | | | | | | | | |
| *Less than once a month* | -0.27 | (-1.45, 0.91) | 0.66 | -0.21 | (-1.17, 0.75) | 0.67 | -0.30 | (-0.78, 0.18) | 0.23 | 0.55 | (0.18, 0.92) | 0.004 |
| *Once a month* | 0.34 | (-0.93, 1.61) | 0.60 | -1.19 | (-2.21, -0.16) | 0.023 | -0.27 | (-1.09, 0.55) | 0.52 | 0.18 | (-0.33, 0.70) | 0.49 |
| *Several times a month* | 0.28 | (-0.89, 1.45) | 0.64 | -0.62 | (-0.15, 0.24) | 0.16 | 0.056 | (-0.47, 0.59) | 0.84 | 0.12 | (-0.27, 0.51) | 0.54 |
| *Several times a week* | 0.022 | (-1.15, 1.19) | 0.97 | 0.10 | (-0.80, 1.01) | 0.82 | 0.32 | (-0.19, 0.84) | 0.22 | 0.44 | (0.024, 0.86) | 0.038 |
| *Everyday* | 0.58 | (-0.60, 1.77) | 0.33 | -0.057 | (-0.89, 0.77) | 0.89 | -0.18 | (-0.74, 0.38) | 0.53 | 0.31 | (-0.11, 0.72) | 0.15 |
